# Peer Review of “Angiotensin Converting Enzyme 1 Expression in the Leukocytes of Adults Aged 64 to 67 Years”

**DOI:** 10.2196/45278

**Published:** 2023-01-20

**Authors:** Heikki Vapaatalo

**Affiliations:** 1 Department of Pharmacology Faculty of Medicine University of Helsinki Helsinki Finland

**Keywords:** aging, angiotensin converting enzyme, lymphocytes, immunosenescence, inflammaging

*This is a peer-review report submitted for the paper “Angiotensin Converting Enzyme 1 Expression in the Leukocytes of Adults Aged 64 to 67 Years.”* [1]

## Round 1 Review

### General Comments

The study is interesting, and the title promises for me more than the manuscript finally contains. The background, question, and the aim are relevant as explained in the Introduction.

The major concerns the small size of the material (6 subjects), the small age difference (64-67 years), and the lack of younger controls.

### Specific Comments

Title: ACE seems better than ACE1; or, does the sophisticated, elegant method include both ACEs? The same should be explained and taken into consideration throughout the text.

Introduction: in the last chapter, the author should explain in more detail how Pawelec et al [2], Alves et al [3], Alves and Bueno [4], and Bueno et al [5] suggest that “ACE1 plays an important role in the aging process.” Does “ACE1 plays” mean, that ACE1 is somehow regulating the aging process or are ACE1 levels changed with age?

Methods: The N value of the subjects should be mentioned here, as well the relation of females and males. Do the authors really regard 64-67 years “older age” nowadays? The study lacks younger controls. Why were the initial assays done many years after the collection of blood samples? Are the samples still useable and not destroyed? Did the subjects have some diseases or were taking drugs because they possibly were from a hospital sample bank? Provide the companies’ details.

Results: “Table 1 shows that older adults…..” The comparison between the present data and historical studies belongs to the Discussion. Also, provide individual ages and genders of the subjects in Table 1. What do *P* values mean here—what is being compared, or are interindividual differences being highlighted in the particular variables? This should be explained. The numbering of tables and the text seems confusing to me. Only 3 tables, but in the text, 4 are mentioned. Table 4 does not exist. It would be good to have a list of abbreviations used in the description of the cell types for an unfamiliar reader.

Discussion: A major part of the discussion deals with previous publications and not meaning or clinical significance of the present findings and comparison between the present and earlier studies. In those previous studies, ACE2 has also has been reported; why is it not studied here? In the limitations paragraph, the authors fairly mention the real problem—the small sample size, and I would like to add a lack of younger subjects. The point regarding the COVID-19 pandemic, seemingly worth mentioning, is too far from this study and unnecessary. Linguistic checking would improve the manuscript.

In summary, I recommend the acceptance of the manuscript for publication after the authors carefully rethink the message of the Results and correct per the minor comments. I hope that in the future, possible age -related correlations to old age of up to >80 years would be possible.

### Decision

Verified with reservations: The content is scientifically sound but has shortcomings that could be improved by further studies and minor revisions.

### Decision Changed

Verified manuscript: The content is scientifically sound, and only minor amendments (if any) are suggested.

## Round 2 Review

I read with pleasure the very detailed answers to my comments. I very warmly recommend the acceptance of this manuscript for publication without any further notes.

### Decision Changed

Verified manuscript: the content is scientifically sound, and only minor amendments (if any) are suggested.

## References

[1] Bueno V, Destro PH, Teixeira D, Frasca D (2023). Angiotensin Converting Enzyme 1 Expression in the Leukocytes of Adults Aged 64 to 67 Years. JMIRx Med.

[2] Pawelec G, Picard E, Bueno V, Verschoor CP, Ostrand-Rosenberg S (2021). MDSCs, ageing and inflammageing. Cell Immunol.

[3] Alves AS, Ishimura ME, Duarte YADO, Bueno V (2018). Parameters of the immune system and vitamin D levels in old individuals. Front Immunol.

[4] Alves A, Bueno V (2019). Immunosenescence: participation of T lymphocytes and myeloid-derived suppressor cells in aging-related immune response changes. Einstein (Sao Paulo).

[5] Bueno V, Sant'Anna Osvaldo Augusto, Lord JM (2014). Ageing and myeloid-derived suppressor cells: possible involvement in immunosenescence and age-related disease. Age (Dordr).

